# VKORC1L1, An Enzyme Mediating the Effect of Vitamin K in Liver and Extrahepatic Tissues

**DOI:** 10.3390/nu10080970

**Published:** 2018-07-26

**Authors:** Julie Lacombe, Mathieu Ferron

**Affiliations:** 1 Integrative and Molecular Physiology research unit, Institut de Recherches Cliniques de Montréal, Montréal, QC H2W 1R7, Canada; julie.lacombe@ircm.qc.ca; 2 Department of Medicine and Molecular Biology Programs of the Faculty of Medicine, Université de Montréal, QC H3C 3J7, Canada; 3 Division of Experimental Medicine, McGill University, Montréal, QC H4A 3J1, Canada

**Keywords:** Vitamin K, vitamin K-dependent carboxylation, coagulation, VKORC1, VKORC1L1, osteocalcin, GGCX, warfarin, vitamin K oxidoreductase

## Abstract

Vitamin K is an essential nutrient involved in the regulation of blood clotting and tissue mineralization. Vitamin K oxidoreductase (VKORC1) converts vitamin K epoxide into reduced vitamin K, which acts as the co-factor for the γ-carboxylation of several proteins, including coagulation factors produced by the liver. VKORC1 is also the pharmacological target of warfarin, a widely used anticoagulant. Vertebrates possess a VKORC1 paralog, VKORC1-like 1 (VKORC1L1), but until very recently, the importance of VKORC1L1 for protein γ-carboxylation and hemostasis in vivo was not clear. Here, we first review the current knowledge on the structure, function and expression pattern of VKORC1L1, including recent data establishing that, in the absence of VKORC1, VKORC1L1 can support vitamin K-dependent carboxylation in the liver during the pre- and perinatal periods in vivo. We then provide original data showing that the partial redundancy between VKORC1 and VKORC1L1 also exists in bone around birth. Recent studies indicate that, in vitro and in cell culture models, VKORC1L1 is less sensitive to warfarin than VKORC1. Genetic evidence is presented here, which supports the notion that VKORC1L1 is not the warfarin-resistant vitamin K quinone reductase present in the liver. In summary, although the exact physiological function of VKORC1L1 remains elusive, the latest findings clearly established that this enzyme is a vitamin K oxidoreductase, which can support γ-carboxylation in vivo.

## 1. Introduction: Vitamin K, Gamma-Carboxylation and Vitamin K Oxidoreductase

Vitamin K is an essential nutrient that is needed to support normal coagulation and to prevent abnormal calcification of arteries and cartilages [1,2]. Clinical studies suggest that vitamin K may also be important to prevent osteoporosis and type 2 diabetes [3,4,5], although the mechanisms of action of vitamin K on bone and glucose metabolism remain unclear. Severe vitamin K deficiency in adults is rare, but is not uncommon in newborns, since vitamin K does not easily cross the placenta and the level of vitamin K is low in breast milk. A reduced level of vitamin K in neonates is a predisposition of the hemorrhagic disease of the newborn, a condition characterized by unexpected bleeding, often with gastrointestinal hemorrhages, ecchymosis and, in many cases, intracranial hemorrhages, which can be life threatening. In most Western countries, a single prophylactic intramuscular injection of vitamin K is administered at birth and greatly reduces the rate of the hemorrhagic disease of the newborn [6].

In vertebrates, the only well-described cellular function of vitamin K is to support γ-glutamyl carboxylation (γ-carboxylation), a posttranslational modification present in some secreted proteins and characterized by the addition of a carboxyl group to specific glutamic acid (Glu) residues, which converts them to γ-carboxyglutamic acid (Gla) residues [2]. Several coagulation factors, produced by the liver, including prothrombin, factor VII, factor IX and factor X, are γ-carboxylated proteins (Gla proteins), and this modification is essential to their function [7], explaining the positive effect of vitamin K on the clotting cascade. Other notable proteins targeted by γ-carboxylation include the matrix Gla protein (MGP), which prevents the calcification of extra-osseous tissues [8], and osteocalcin, a bone-derived hormone regulating glucose and energy metabolism in its undercarboxylated form [9,10]. γ-carboxylation is completed in the endoplasmic reticulum (ER) by the γ-glutamyl carboxylase (GGCX), an enzyme requiring vitamin K hydroquinone as a co-factor, which is then oxidized to vitamin K 2,3-epoxide during the carboxylation reaction [11]. Vitamin K is recycled in a two-step reduction process, wherein vitamin K 2,3-epoxide is first converted to vitamin K quinone, which is then reduced to vitamin K hydroquinone. In physiological conditions, these two steps were thought to be catalyzed by a single enzyme, the vitamin K oxidoreductase (VKOR or VKORC1), which is encoded by the gene *VKORC1* [12,13,14,15,16]. Together, the enzymatic activities of GGCX and VKORC1 form the vitamin K cycle and warfarin, an anticoagulant used by millions of people (see Section 11), preventing the γ-carboxylation of coagulation factors by blocking the vitamin K cycle through the direct inhibition of VKORC1 [12,17]. 

## 2. VKOR Homologues Are Present in Metazoans, Protists, Bacteria and Plants 

VKOR and GGCX homologues are found in the genome of almost all metazoans, including insects (*D. melanogaster*) and placozoa (*T. adhaerens*), with the remarkable exception of nematodes, which apparently lost all the components of the vitamin K cycle [18,19]. It is noteworthy that VKOR homologues are also found in some protists, bacteria and plant species, which are nevertheless lacking GGCX. These phylogenic studies suggest that the ancestral VKOR enzyme appeared during evolution before GGCX, potentially fulfilling a function not linked to protein carboxylation. Supporting this idea, bacterial VKOR homologues have been shown to be involved in disulfide bond formation in the periplasm [20,21]. It is not clear, however, whether the natural substrate of bacterial VKORs is vitamin K_2_ (menaquione), which is synthesized by several bacteria, or other bacterial quinones, such as ubiquinone (coenzyme Q_10_). Interestingly, a single bacterial VKOR homologue, isolated from *Mycobacterium tuberculosis*, was found to be able to support vitamin K-dependent carboxylation in mammalian cells lacking endogenous VKORC1 activity [22]. In addition, bacterial VKOR homologues have been shown to be able to reduce vitamin K to vitamin K hydroquinone in vitro, however, they do not seem to reduce vitamin K 2,3-epoxide in the same assay. This is in line with the observation that vitamin K 2,3-epoxide is not present in bacteria [23]. It is therefore likely that bacterial VKOR homologues function in bacteria as vitamin K quinone reductases, but not as vitamin K oxidoreductase. The putative ubiquinone reductase activity of these enzymes remains speculative, since it has not been tested experimentally.

## 3. VKORC1L1 Is A Vertebrate Paralog of VKORC1

**Figure 1:**
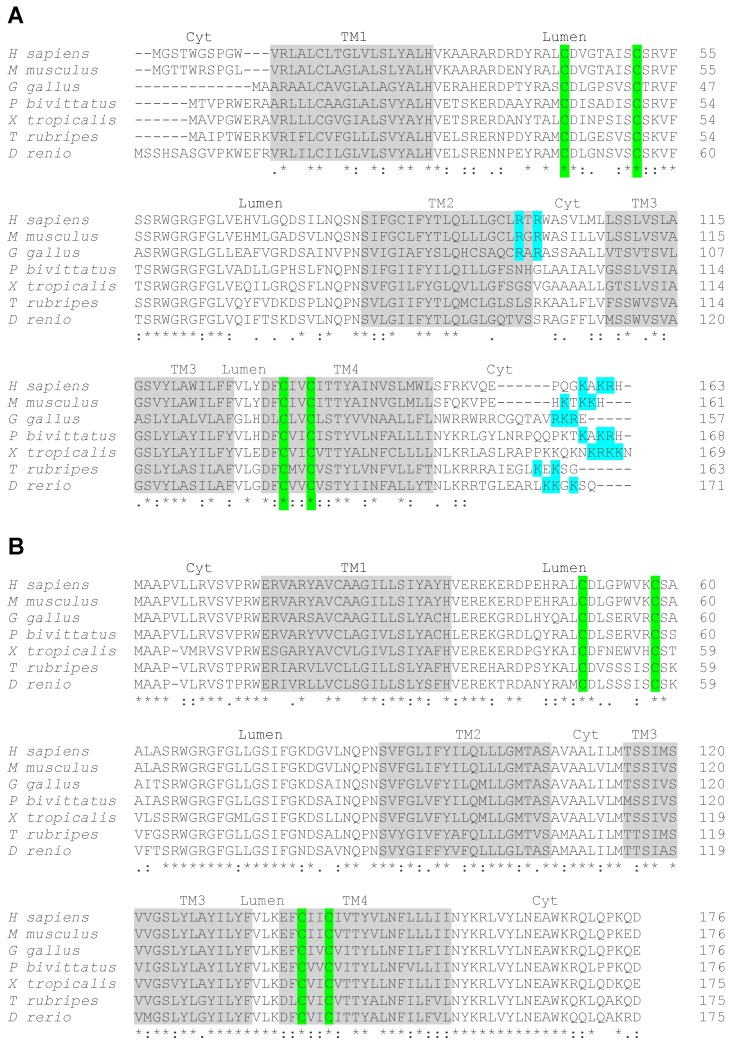
Sequence alignment of vertebrate vitamin K oxido reductases. Vertebrate VKORC1 (**A**) and VKORC1L1 (**B**) sequences from humans (*H. sapiens*), mice (*M. musculus*), chickens (*G. gallus*), Burmese pythons (*P. bivittatus*), clawed frogs (*X. tropicalis*), Japanese puffer fish (*T. rubripes*) and zebrafish (*D. rerio*). The predicted four transmembrane domains (TM 1-4) are highlighted in grey. The localization of the different loops in the cytosol (Cyt) or in the lumen (Lumen) is indicated above the sequences. The conserved four cysteine residues involved in the enzymatic activity of the proteins are highlighted in green. In VKORC1 the c-terminus di-lysine motif and the internal di-arginine motif, potentially involved in ER retention, are highlighted in blue. Consensus symbols are included below the alignment. A single asterisk (*) indicates a fully conserved residue; a colon (:) indicates a strongly conserved residue; and a period (.) indicates moderate or weak conservation.

Vertebrates, including fishes, rodents and humans, possess two genes encoding VKORs, VKORC1, and a paralog, named VKORC1-like 1 (VKORC1L1) [12,19]. Phylogenic analyses suggest that these two paralogs derive from a single ancestral *Vkor* gene and that the duplication event that generated two separate genes has occurred in a primitive vertebrate at the origin of the urochordate and vertebrate lineages [19]. Interestingly, the protein sequence alignment of VKORC1 and VKORC1L1 homologues from a range of vertebrate species, including mammals (human and mouse), birds (chickens), reptiles (pitons), amphibians (frogs) and fish (Japanese puffer fish and zebrafish), reveals a remarkable difference in their respective degree of sequence conservation (Figure 1A,B). Only 49 out of the 163 amino acids (~30%) of human VKORC1 are conserved throughout the various vertebrate homologues (Figure 1A). In contrast, 104 amino acids out of 176 of human VKORC1L1 (~60%) are fully conserved in all the tested homologues (Figure 1B). This analysis is consistent with previous, more extensive phylogenic studies [19,24] and suggests that VKORC1 was more free to diverge than VKORC1L1, following gene duplication, for reasons that remain unclear. One proposed hypothesis is that VKORC1L1 has retained the original housekeeping functions of the ancestral VKOR, while VKORC1 has diverged to acquire a novel, more specific function in supporting robust vitamin K-dependent carboxylation in the liver [24].

## 4. Structure of VKORC1 and VKORC1L1

The amino acid sequences of VKORC1 and VKORC1L1 are characterized by the presence of predicted transmembrane helixes and of 4 conserved cysteine residues (highlighted in grey and in green in Figure 1A,B, respectively). The crystal structure of the cyanobacterium *Synechococcus* sp. VKOR homologue shows that it folds around a four transmembrane helix (TM) bundle, which contains the catalytic core [15,16], with the n-and c-terminus of the protein located in the cytosol. Based on this model, in human VKORC1, the cysteine residues, contained in the TM4 (Cys132 and Cys135) and in the loop between TM1 and TM2 (Cys43 and Cys51), are localized in or close to the endoplasmic reticulum lumen (Figure 1A). The two cysteines, embedded in the TM4 have been shown to form the enzymatic redox center and are essential for both the vitamin K quinone reductase and the vitamin K 2,3-epoxide reductase activity [25,26]. The two loop cysteines have been shown to serve as shuttles to transfer electrons from a redox partner, present in the ER lumen, to the cysteines of the redox center. This redox partner has been proposed to be an ER membrane-anchored Trx-like protein, which could be TMX, TMX4 or ERp18 [27].

**Figure 2:**
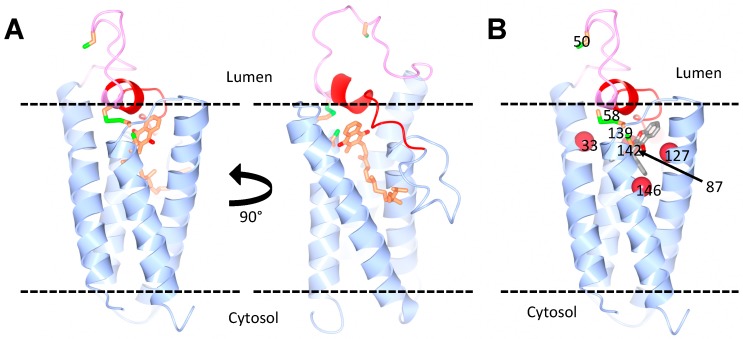
Predicted structure of human VKORC1L1. (**A**) Homology models of VKORC1L1, bound to vitamin K, based on the structure of the bacterial VKOR homologue. The vitamin K molecule is shown in orange. Two views of the model are shown with a 90° horizontal rotation. (**B**) Model of VKORC1L1 bound to warfarin. The warfarin-binding pocket (grey) is surrounded by residues (red spheres) that, when mutated, cause a strong resistance in both VKOR1L1 and VKORC1 (Ala33, Asn87, Leu127 and Y146 in VKORC1L1). The cap domain (red) and the luminal loop (pink) are also highlighted. The conserved cysteines (green and orange) are shown: Cys58 and Cys139 as disulfide, and Cys50 and Cys142 as free. Courtesy of Dr. Weikai Li, Washington University in St. Louis.

Whether human VKORC1 is organized in a four-TM structure, like the bacterial VKOR homologue, has been controversial, since the biochemical analysis of human VKORC1 topology generated conflicting conclusions, supporting either a four-TM [28,29,30,31,32,33] or a three-TM model [17,27,34]. Indeed, another model has been proposed for human VKORC1, in which the protein contains only three transmembrane helixes and where Cys43 and Cys51 are localized in the cytosol. A critical evaluation of the technical details, which could explain the discrepancy between some biochemical data and the structural biology predictions, has been published recently [35]. We note, most importantly, that the study of intact human VKORC1, using live cell cysteine labeling in combination with mass spectrometry, convincingly showed that a major fraction of Cys43, Cys51, Cys132 and Cys135 is oxidized in living cells, strongly suggesting that they are all located in the oxidative ER lumen [17]. Together with the structural data, these results support the four-TM model for human VKORC1 [35]. Importantly, biochemical and structural modeling predict that human VKORC1L1 is also organized as a four-TM protein (Figure 2A) and that the two loop cysteine residues of VKORC1L1 are essential for its activity [30,33].

## 5. Subcellular Localization of VKORC1 and VKORC1L1

It was at first assumed that the two VKOR paralogs are both localized in the ER membrane, although recent data suggest that they have different subcellular localization in mammalian cells. In osteoblasts and in HEK293 human embryonic kidney cells, VKORC1 extensively co-localizes with ER markers, which is consistent with the observation that vitamin K-dependent carboxylation occurs in the ER [33,36,37] and that vitamin K oxidoreductase activity concentrates in the microsomal fraction in hepatocytes [38]. VKORC1L1 is also found in the ER, but significant non-ER localization is also apparent for this protein [33,36], suggesting that VKORC1L1 may possess different function(s) in other organelles. 

The characteristic ER distribution of VKORC1 can be explained by the presence, in its c-terminal cytosolic tail, of a di-lysine ER retention signal (“KAKRH” in human VKORC1) [33]. Most vertebrate paralogs of VKORC1 also possess a di-lysine or a di-lysine-arginine motif in their c-terminus (Figure 1A, in blue). Mutating the lysine into alanine residues or masking the c-terminal motif with an artificial sequence, mislocalizes VKORC1 and significantly decreases the capacity of VKORC1 to support vitamin K-dependent carboxylation in a cell-based assay [33,37]. A second ER retention signal, consisting of a di-arginine motif (“RTR”) between TM2 and TM3, was also shown to influence the localization of human VKORC1 [37]. Neither of these motifs is present in the sequence of mammalian VKORC1L1 [33] (Figure 1B), suggesting that this protein sub-cellular localization differs from the one of VKORC1 because it lacks an ER-retention signal.

## 6. Evidence That VKORC1L1 Supports Gamma-Carboxylation in Cell Culture Models 

Following its identification, two key biological questions concerning VKORC1L1 were whether this protein is a functional vitamin K oxidoreductase and, if so, whether it can support γ-carboxylation. In dithiothreitol (DTT)-driven VKOR activity assays, human and rat VKORC1L1 were found to be able to support vitamin K 2,3-epoxide to vitamin K reduction almost as efficiently as VKORC1 [39,40]. Using independently developed cell culture assays, allowing the quantitative measurement of the γ-carboxylation of a reporter protein (i.e., Factor IX or a Factor X-protein C chimera), two groups have shown that in HEK293 cells, the inactivation of both VKORC1 and VKORC1L1 is necessary to abolish vitamin K-dependent γ-carboxylation [30,33,41,42]. Moreover, in HEK293 cells lacking the two VKORs, the reintroduction of VKORC1L1 alone is sufficient to fully restore vitamin K epoxide-dependent γ-carboxylation [33,42,43]. These experiments suggest that VKORC1L1 can support vitamin K-dependent γ-carboxylation in the absence of VKORC1 in this particular cell line. This may not be true for all cell types, since one study has shown that the inactivation of VKORC1 was sufficient to impair the γ-carboxylation of the bone Gla protein osteocalcin in primary mouse osteoblasts in cell culture [36]. That the inactivation of both VKORC1 and VKORC1L1 did not further impact upon osteocalcin γ-carboxylation suggests that, in some conditions, VKORC1L1 may not be able to compensate for the absence of VKORC1. 

## 7. Expression Pattern of *Vkorc1* and *Vkorc1l1* in Adult Tissues

Given the in vitro data, suggesting that both VKORC1 and VKORC1L1 equally support γ-carboxylation, one possible explanation for the existence of these two paralogs in vertebrates could be that VKORC1 and VKORC1L1 are differentially expressed and hence support γ-carboxylation in distinct tissues. To address this possibility, a few studies have examined the expression pattern of the two VKOR-encoding genes in rodent and human tissues. An extensive characterization of *Vkorc1* and *Vkorc1l1* expression in 29 different adult mouse tissues suggests that *Vkorc1* is predominantly expressed in liver and in exocrine tissues, such as the mammary gland, salivary gland and prostate [44]. *Vkorc1l1* levels are the highest in the brain and the lowest in liver, but it is important to note that, even in the brain, *Vkorc1l1* is expressed at either an equal or lower level than *Vkorc1*. Vitamin K oxidoreductase activity, assessed using the DTT-driven assay, was detected in microsomal preparation in virtually all tissues tested with high values in the liver, pancreas, ovary and uterus, and low levels in the brain and gastrointestinal track [44]. Another study examined the expression of these two genes in five adult rat tissues and also observed that *Vkorc1* is about 10 times more expressed than *Vkorc1l1* in the liver, but that the two genes are expressed at similar levels in the lung, kidney, brain and testis [40]. Interestingly, the same study shows that, although VKOR activity is reduced by more than 30-fold in *Vkorc1^−/−^* mouse livers, there is still ~35% of detectable VKOR activity in *Vkorc1^−/−^* testis, supporting the presence of a second VKOR enzyme in this tissue [40]. Importantly, *Vkorc1l1* expression was not changed in *Vkorc1^−/−^* tissues or in HEK293 cells lacking *Vkorc1l1* [40,41], implying that the transcription of these two genes is independently regulated. Altogether, these various studies clearly established that VKORC1 is the predominant paralog in the adult liver. Importantly, however, no tissue or cell type has yet been identified, in which *Vkorc1l1*, but not *Vkorc1*, is expressed, since both *Vkorc1* and *Vkorc1l1* are broadly and mostly equally expressed in extrahepatic tissues. Based on its expression pattern, it therefore remains difficult to predict any unique physiological function for VKORC1L1. 

It was suggested that, in HEK293 cells, VKORC1L1 could protect cells from oxidative stress in a vitamin K-dependent manner [39]. However, the inactivation of VKORC1L1 does not impact HEK293 cell survival in culture [41,42], and no obvious phenotype potentially associated with increased oxidative stress has been detected in *Vkorc1l1^−/−^* mice, which are viable and develop normally in comparison to wild-type mice [36,45]. Overall, the genetic data are not in support of VKORC1L1 having a major role in protecting from oxidative stress, at least under physiological conditions. 

## 8. Evidence That VKORC1L1 Supports Gamma-Carboxylation In Vivo 

Consistent with the key function played by the vitamin K cycle in coagulation, homozygous mutations in *GGCX* and *VKORC1* in humans have been found to cause combined deficiency of VK-dependent clotting factors 1 and 2, respectively [12,46,47]. These rare syndromes are characterized by very low circulating level of all the vitamin k-dependent coagulation factors and intracranial hemorrhages in early infancy. Similarly, the deletion of either *Ggcx* or *Vkorc1* in mice resulted in perinatal or postnatal death caused by bleeding defects [36,48,49]. Based on the observation that VKORC1 deficiency in mice and humans is sufficient to cause the undercarboxylation of clotting factors and hemorrhages, it was initially concluded that VKORC1L1 could not support vitamin K-dependent γ-carboxylation in the absence of VKORC1, at least in the liver [48]. In support of this notion, as mentioned above, it was also observed that *Vkorc1* mRNA is 10 to 20 times more abundant than *Vkorc1l1* mRNA in the adult liver [40,44]. However, an important difference between *Ggcx^−/−^* and *Vkorc1^−/−^* mice was noted, which questioned this view: While the *Ggcx^−/−^* mice, similarly to the prothrombin-deficient mice [50], die during late embryogenesis or immediately after birth from intra-abdominal hemorrhages [49], most of the *Vkorc1^−/−^* mice survive for at least one week following birth (P7) before dying from hemorrhages [48]. The survival of *Vkorc1^−/−^* mice on a pure C57Bl/6J background was reanalyzed and it was found that the VKORC1 deficiency was compatible with postnatal survival for more than one week after birth, but that all *Vkorc1^−/−^* animals died before 20 days after birth, with a median survival of 9.5 days. In addition, prothrombin and GGCX are γ-carboxylated in e18.5 *Vkorc1^−/−^* embryos and newborns, but not in one-week-old *Vkorc1^−/−^* pups [45]. These observations suggested the existence of a second enzyme, which could support vitamin K-dependent γ-carboxylation in the liver during embryogenesis and perinatally. 

Therefore, further genetic experiments were performed to assess the possible redundant action of VKORC1 and VKORC1L1 on vitamin K-dependent γ-carboxylation, coagulation and postnatal survival. Strikingly, mice in which one allele of *Vkorc1l1* is inactivated on a *Vkorc1^−/−^* background (i.e., *Vkorc1^−/−^*; *Vkorc1l1^+/−^*) die from hemorrhages within 2 days after birth, while mice lacking all four alleles of VKORs (i.e., *Vkorc1^−/−^*; *Vkorc1l1^−/−^*) die at birth from uncontrolled bleeding [45]. Hence, VKORC1L1 protect mice from hemorrhages and premature death in the absence of VKORC1. γ-carboxylation is present in *Vkorc1^−/−^* embryos and newborn livers, but is greatly reduced or absent at the same age in *Vkorc1^−/−^*; *Vkorc1l1^+/−^* and *Vkorc1^−/−^*; *Vkorc1l1^−/−^* animals, respectively, clearly demonstrating that VKORC1L1 supports γ-carboxylation in the absence of VKORC1. Finally, a VKOR enzymatic assay performed on control, *Vkorc1^−/−^* and *Vkorc1^−/−^*; *Vkorc1l1^+/−^* newborn livers revealed that VKORC1L1 contributes to less than 1% of vitamin K 2,3-epoxide reduction in this tissue [45]. This low level is nevertheless sufficient to support enough γ-carboxylation to prevent bleeding until one week after birth.

## 9. Developmental Regulation of the Vitamin K Cycle in the Liver

**Figure 3:**
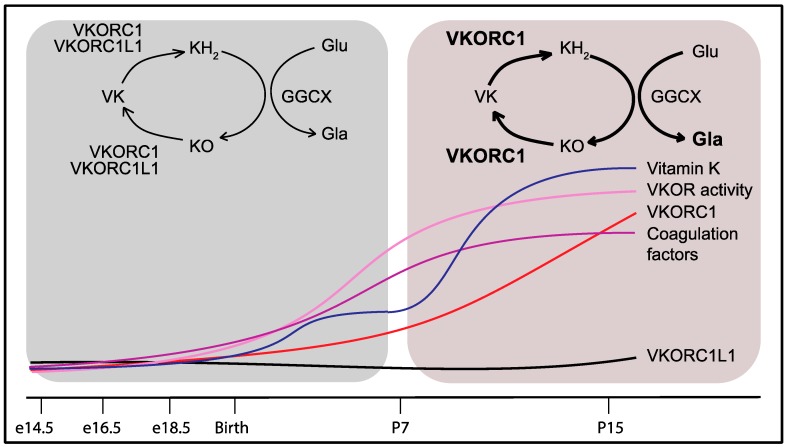
Developmental regulation of VKORC1 and of the vitamin K-dependent coagulation factors in the liver explains the partial redundancy of VKORC1L1. Around birth, VKORC1 and VKORC1L1 both support γ-carboxylation and hemostasis. Beyond P7, VKORC1 expression and activity increase in the liver with the level of vitamin K concomitantly rising in the bloodstream to handle the growing demand for coagulation factor γ-carboxylation. The lower expression levels of VKORC1L1 in the liver after P7 can no longer compensate for the absence of VKORC1 activity. VKORC1: Vitamin K oxidoreductase; VKORC1L1: VKORC1-like 1; VK: Vitamin K; KH_2_: Vitamin K hydroquinone; KO: Vitamin K 2,3-epoxide; GGCX: γ-glutamyl carboxylase; Glu: Glutamic acid residue; Gla: γ-carboxyglutamic acid residue.

If VKORC1L1 can support vitamin K reduction and γ-carboxylation in the liver during the prenatal and perinatal period, why does it fail to compensate for the absence of VKORC1 after one week following birth? One plausible answer to this question was that VKORC1 and VKORC1L1 are differentially expressed during development. Gene expression analysis revealed that *Vkorc1* and *Vkorc1l1* expressions in the liver remain stable and comparable between the embryonic day 14.5 (e14.5) and birth (P0). However, the expression of *Vkorc1* increases 10–20 fold between P0 and postnatal day 15 (P15), while the expression of *Vkorc1l1* remains at the same level [45]. Liver VKOR enzymatic activity also increases robustly after birth, suggesting that the high postnatal level of VKOR activity can be mostly ascribed to VKORC1, as previously suggested by the very low level of VKOR activity in adult *Vkorc1^−/−^* livers [40]. The strong postnatal increase in VKORC1 is accompanied by the rapid and robust up-regulation of the expression of the gene encoding for the vitamin K-dependent coagulation factors, prothrombin, factor VII, factor IX and factor X. Moreover, the expression of the γ-carboxylase (*Ggcx*) and the presence of Gla proteins rise in the liver between P0 and P15, while the plasma level of carboxylated prothrombin is ~10-fold higher in P15 than in e16.5 mice [45]. Altogether, these results indicated that the presence of functional coagulation factors is significantly increased following birth and that this augmentation is sustained by a concerted up-regulation in the liver of the genes encoding these factors and only one of the two VKOR-encoding genes, i.e., VKORC1 (Figure 3). The transgenic overexpression of VKORC1L1, under the control of the human APOE promoter (*APOE-Vkorc1l1*) in the liver of *Vkorc1^−/−^* mice, was sufficient to restore γ-carboxylation and normal coagulation. In fact, *Vkorc1^−/−^*; *APOE-Vkorc1l1* mice survive into adulthood, are fertile and do not display any apparent phenotype. Hence, VKORC1L1, when expressed at a sufficient level, can sustain vitamin K-dependent carboxylation in the adult liver and prevent bleeding and lethality [45]. 

These results provide a biological explanation for 30-year-old clinical observations, showing that vitamin K-dependent coagulation factor activities and level in plasma are, on average, 75% lower in normal human fetuses compared to adults [51,52]. Other early clinical studies, establishing that, following birth, the levels of prothrombin, factor VII, factor IX and factor X rapidly increase in serum, reaching adult levels after 2 to 6 months following birth in humans [53], are also in line with our results. One question that remains unanswered is why clotting factors and the carboxylation machinery need to be up-regulated only after birth? A possible answer could be that fetuses and embryos are less prone to injuries and hence to bleeding than newborns and adults. Vitamin K-dependent carboxylation may also be involved in embryonic development, since about one half of the embryo lacking the γ-carboxylase (*Ggcx^−/−^*), prothrombin (*F2^−/−^*) or factor X (*F10^−/−^*) die during mid-gestation [49,50,54]. Since minimal level of maternal vitamin K crosses the placenta [55], fetuses have a very limited supply of vitamin K. Therefore, the lower expression of vitamin K-dependent coagulation factors, VKORC1 and GGCX in the liver during embryogenesis may be required for the preferential usage of vitamin K by extra-hepatic tissues involved in development. 

## 10. Partial Redundancy between VKORC1 and VKORC1L1 in Osteoblasts during the Perinatal Period 

**Figure 4:**
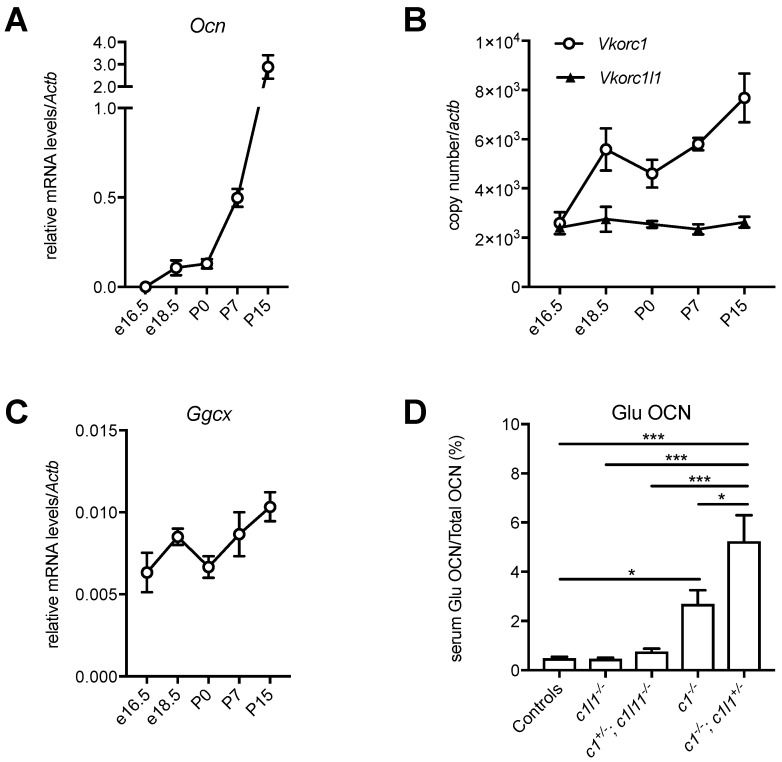
Developmental regulation of the vitamin K cycle in bones. (**A**–**C**) Calvaria from e16.5, e18.5, P0, P7 and P15 C57BL/6J mice were harvested, and the total RNA was extracted (mean ± standard error of the mean (SEM), *n* = 3). (**A**) Relative mRNA levels of the gene encoding osteocalcin (*Ocn*) were measured by quantitative PCR (qPCR) and normalized to the actin-β gene *Actb*. (**B**) *Vkorc1* and *Vkorc1l1* gene expressions were measured by qPCR and normalized to *Actb*. The copy number was quantified using plasmid DNA, containing either *Vkorc1* or *Vkorc1l1* cDNA as a standard. (**C**) Relative mRNA levels of *Ggcx* were measured by qPCR and normalized to *Actb*. (**D**) Decarboxylated osteocalcin (Glu OCN) and total osteocalcin (total OCN) serum levels were measured in newborn mice (P0), for the indicated genotypes (*c1*: *Vkorc1*; *c1l1*: *Vkorc1l1*), using previously described assays [59] and the level of Glu OCN, expressed as a percentage of total OCN (mean ± standard error of the mean (SEM), *n* = 4–18, * *p* < 0.05, *** *p* < 0.001).

The finding that VKORC1 and VKORC1L1 can redundantly support vitamin K-dependent carboxylation in the liver during embryogenesis and around birth, but not in adults, raises the question of whether a similar redundancy exists in any other tissue. Osteoblast, the bone forming cell, is another cell type in which vitamin K-dependent carboxylation plays an important role through the regulation of the bone-derived hormone, osteocalcin [9,10,56]. It was previously shown that the conditional inactivation of *Vkorc1* in differentiated osteoblasts using *OC-Cre* (i.e., *Vkorc1^fl/fl^*; *OC-Cre*) is sufficient to prevent the γ-carboxylation of osteocalcin in adult mice [36]. However, whether this is also true in embryos or newborn mice, is unknown. Interestingly, osteocalcin serum level, which is low during embryogenesis (i.e., ~10–20 ng/mL between e14.5 and e17.5), increases rapidly after birth, reaching values of ~150 ng/mL at P0, ~250 ng/mL at P10 and >1000 ng/mL at P15 [57,58]. We could show that this increase is explained by a gradual up-regulation of the expression of the gene encoding for osteocalcin (*Ocn*) in bones during the perinatal period (Figure 4A). 

These observations suggest that, similarly to the liver, the components of the vitamin K cycle might be regulated during the development of bones to sustain the increasing demand for osteocalcin carboxylation. The mRNA expression level of *Vkorc1*, *Vkorc1l1* and *Ggcx* was therefore measured in calvaria bones isolated from embryos at different developmental stages (e16.5 and e18.5), in newborns (P0), P7 and P15 mice. While *Vkorc1l1* expression remains stable in bones, in both the prenatal and postnatal periods, *Vkorc1* expression increases sharply around the perinatal period (e18.5 and P0), and *Ggcx* expression increases postnatally (Figure 4B,C). Since both *Vkorc1* and *Vkorc1l1* are expressed in bones around birth (Figure 4B), this suggested that these two genes could redundantly regulate vitamin K-dependent carboxylation of osteocalcin in osteoblasts during the perinatal period. Serum measurement of the carboxylation status of osteocalcin in newborn mice with various *Vkorc1* and *Vkorc1l1* genotypes supports this hypothesis. As expected, the serum level of decarboxylated osteocalcin (Glu OCN) is higher in *Vkorc1^−/−^* newborns compared to controls. Nevertheless, Glu OCN is further increased in *Vkorc1^−/−^*; *Vkorc1l1^+/−^* newborns (Figure 4D), indicating that VKORC1L1 can support carboxylation to some extent in osteoblasts in the absence of VKORC1 around birth. Altogether, these additional findings support the idea that VKORC1 and GGCX are also developmentally regulated in bones and that VKORC1L1 can compensate for the absence of VKORC1, but only during the prenatal and perinatal period, when the need for vitamin K-dependent carboxylation is lower. These observations suggest that the expression of *Vkorc1* and *Ggcx* are coupled with the expression of particular vitamin K-dependent proteins (i.e., osteocalcin in bones and coagulation factors in the liver), implying the existence of tissue-specific transcriptional mechanisms that regulate both the expression of the carboxylation machinery and the proteins they modify. *Vkorc1l1* is expressed at a constant level throughout the development both in liver [45] and bones (Figure 4B), and is detected at medium to low levels in most adult tissues [44]. Those are two features often characterizing housekeeping genes, and it is therefore tempting to hypothesize that VKORC1L1 is responsible for supporting the basal level of vitamin K reduction in most tissues, while VKORC1 has evolved to be temporally regulated and highly expressed in cell types, such as hepatocytes and osteoblasts, where a very high demand for carboxylation is needed after birth. 

## 11. VKORC1L1 Is Less Sensitive to Warfarin Than VKORC1 In Vitro

Oral anticoagulants, or vitamin K antagonists, are a class of drug that targets VKORC1, used to treat and prevent thromboembolism, and include warfarin, acenocoumarol, phenprocoumon and fluindione [60]. In addition to those drugs, several derivatives of warfarin, called superwarfarins, have been developed to serve as rodenticide. Superwarfarins, such as bromadiolone and brodifacoum, have a much higher affinity to VKORC1, a tendency to accumulate in tissues and a very long half-life in vivo, compared to warfarin and other therapeutic vitamin K antagonists [61]. Whether VKORC1 and VKORC1L1 are equally sensitive to warfarin and other vitamin K antagonists was unknown, until a series of recent studies began to address this question using in vitro and cell based assays.

In DTT-driven VKOR enzymatic assay on microsome extracts of the yeast, *Pichia pastoris*, heterologously overexpressing either one of the two VKOR paralogs, VKORC1L1 appears to be, on average, 30 to 50-fold less sensitive to warfarin than VKORC1 [40]. Another study has shown, using the same DTT-driven VKOR assay, that VKORC1L1 is only 2 times less sensitive to warfarin compared to VKORC1 in HEK293 crude cell extracts, overexpressing these proteins [39]. 

Cell-based assays taking advantage of the measurement of the carboxylation level of a reporter protein, secreted by HEK293 cells, have also been used to compare the relative sensitivity of VKORC1 and VKORC1L1 to warfarin and other vitamin K antagonists. Using HEK293 cells lacking either VKORC1 or VKORC1L1, a first study determined the IC_50_ of these two enzymes for warfarin and several other vitamin K antagonists, including some superwarfarins, by measuring the release of active factor IX by the cells [42]. It was found that VKORC1 is about 13 times more sensitive to warfarin, and 50 times more sensitive to fluidione, than VKORC1L1. In contrast, the two paralogs appear to be equally sensitive to the bromadiolone and brodifacoum rodenticides. One limitation of this study is the use of *Vkorc1*^−/−^ and *Vkorc1l1*^−/−^ single knockout cells to assess the relative sensitivity of each enzyme to these various inhibitors without measuring the relative expression level of VKORC1 and VKORC1L1 proteins in HEK293 cells. Indeed, a different amount of one or the other could potentially impact the observed IC_50_ values, and a recent paper suggested that the VKORC1L1 protein is expressed at a much lower level than VKORC1 in HEK293 cells [62]. A second study compared the sensitivity to warfarin of VKORC1, or VKORC1L1, re-expressed in HEK293 cells lacking both VKOR paralogs, by measuring the carboxylation status of a factor IX-protein C chimeric protein, expressed in the same cells [41,43]. In this assay, and in comparison to VKORC1, VKORC1L1 was found to be 25-fold more resistant to warfarin, which is more consistent with the in vitro observation cited above [40].

Czogalla et al. shows that the difference in the sensitivity of the two enzymes lies within the ER loop between TM1 and TM2. Indeed, swapping the amino acids Lys30 to Ser79 in VKORC1 with the corresponding amino acids (Glu37 to Pro86) in VKORC1L1 is sufficient to confer warfarin resistance to VKORC1 and to make VKORC1L1 more sensitive [42]. Shen et al. further show that the amino acids, Glu37 and His46, in VKORC1L1, which correspond to Lys30 and Tyr39 in VKORC1, are responsible for the different warfarin sensitivity of these two paralogs. Specifically, Glu37Lys and His46Tyr mutations are sufficient to increase VKORC1L1 warfarin sensitivity to levels observed for VKORC1, while the converse mutations in VKORC1, i.e., Lys30Glu and Tyr39His, confer partial warfarin resistance [43].

The first group also concluded, based on in silico modeling, that the location of the warfarin binding site is different in the two proteins and that three arginine residues (Arg38, Arg42 and Arg68) in VKORC1L1 are required for the inhibition by warfarin [42]. Warfarin resistance is a well-known phenomenon in patients undergoing anticoagulant therapy and in rodent populations that have been exposed to warfarin or its derivatives. Several mutations and variants in VKORC1 have been identified as being responsible for warfarin resistance in humans or rodents [12,63,64,65]. Interestingly, many of these resistance mutations occur in amino acids that are conserved between VKORC1 and VKORC1L1. Shen et al. elegantly show that introducing in VKORC1L1 mutations, which were previously shown to confer strong warfarin resistance to VKORC1, also causes strong warfarin resistance in VKORC1L1. They further show that another group of mutations causes weak warfarin resistance in both VKORC1 and VKORC1L1 [43]. According to the crystal structure of the bacterial VKOR homologue, the mutations confer a high warfarin resistance cluster around the putative warfarin-binding site in both VKORC1 and VKORC1L1, a region which overlaps with their active sites (Figure 2B). Hence, in contrast to Czogalla et al. [42], the study by Shen et al. [43] suggests that the two paralogs bind warfarin at the same site.

The stronger resistance to warfarin of VKORC1L1 may protect extra-hepatic tissues from warfarin therapy, since VKORC1 and VKORC1L1 are expressed at a comparable level outside the liver [40,44]. A single nucleotide polymorphism (SNP) in human VKORC1L1, which convert glutamic acid 41 into a lysine, reduces the resistance to warfarin of this enzyme by 80% [43]. Therefore, it is possible that, in individuals carrying this SNP or other yet-to-be-identified warfarin-sensitizing variants, the protective effect of VKORC1L1 in extra-hepatic tissues is lost and vitamin K antagonist therapy could have undesirable side effects, such as increasing the risk of vascular calcification or osteoporosis [66,67,68]. Following this fascinating molecular work, further genetic and clinical studies will be needed to determine whether VKORC1L1 genotypes truthfully influence the adverse effects of warfarin therapy.

## 12. VKORC1L1 Is Not the Liver Warfarin Resistant Vitamin K Quinone Reductase

The reduction of vitamin K to vitamin K hydroquinone is also catalyzed in the liver and in HEK293 cells by a yet-to-be-identified warfarin resistant quinone reductase [62,69,70]. This “antidotal enzyme” allows the production of γ-carboxylated coagulation factors in the presence of otherwise lethal doses of warfarin if large amounts of vitamin K_1_ quinone (i.e., phylloquinone) are concomitantly injected into the animals or provided in the culture media. This unidentified quinone reductase also explains why vitamin K can rescue the bleeding defect of *Vkorc1*^−/−^ mice [48] and of human patients suffering from a multiple coagulation factor deficiency, secondary to a mutation in *VKORC1* or to warfarin overdosing [46]. 

**Figure 5:**
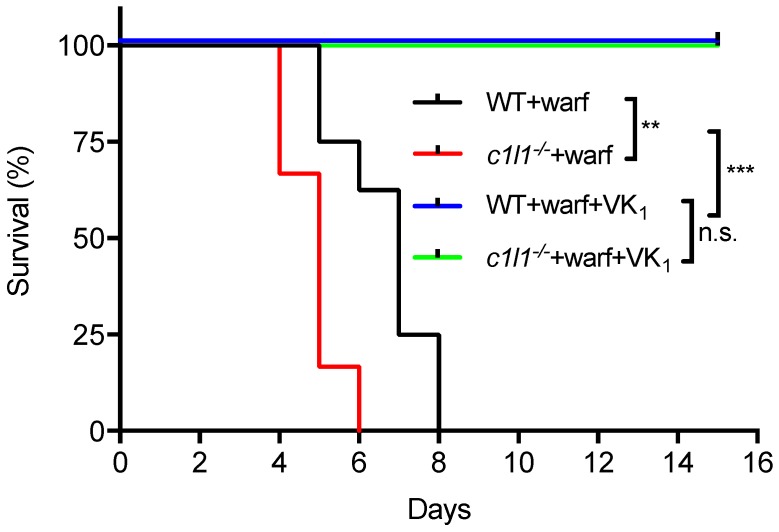
Genetic evidence that VKORC1L1 is not the warfarin resistant vitamin K quinone reductase present in the liver. Kaplan-Meier survival curves were determined for WT (*n* = 8) and *Vkorc1l1*^−/−^ (*c1l1*; *n* = 6) mice, treated with either warfarin alone (150 mg/kg/day) or a combination of warfarin (warf; 150 mg/kg/day) and vitamin K_1_ (VK_1_; 15 mg/kg/day) for a maximum of 15 days (Mantel-Cox test, ** *p* < 0.01 and *** *p* < 0.001). WT: Wild-type; *c1l1*: *Vkorc1l1*; Warf: Warfarin; VK_1_; Vitamin K_1_.

As detailed above, recent findings established that VKORC1L1 is able to support vitamin K quinone reduction and γ-carboxylation in vivo at least in the absence of VKORC1. Moreover, as explained in the previous section, VKORC1L1 is much less sensitive than VKORC1 to warfarin inhibition in vitro and in cell-based assays. These observations raised the possibility that VKORC1L1 could be the liver enzyme capable of reducing vitamin K to vitamin K hydroquinone when VKORC1 is inactivated pharmacologically. This was formally tested genetically in mice. To that end, wild-type or *Vkorc1l1*^−/−^ mice were injected daily with either a lethal dose of warfarin (150 mg/kg/day) or with the same dose of warfarin in combination with a high dose of vitamin K_1_ (15 mg/kg/day), which was previously shown to rescue the warfarin anticoagulant effect in rats [71,72]. As expected, wild-type mice receiving warfarin alone died within 8 days following the initiation of the treatment (median survival of 7 days), mostly from intestinal and subcutaneous hemorrhages (not shown). In contrast, the wild-type mice treated with both warfarin and vitamin K_1_ all survived for more than 15 days (Figure 5). *Vkorc1l1*^−/−^ mice, treated with warfarin and vitamin K_1_, also survived without hemorrhaging. This result indicates that VKORC1L1 is not the warfarin resistant vitamin K quinone reductase present in the liver. Interestingly, *Vkorc1l1*^−/−^ mice receiving warfarin only died earlier than wild-type mice, i.e., within 6 days of the initiation of the treatment (median survival of 5 days; *p* = 0.006). The accelerated death of the *Vkorc1l1*^−/−^ animals suggests that VKORC1L1 is indeed less sensitive to warfarin inhibition in vivo and can partially protect the mice in which VKORC1 is pharmacologically inhibited. Moreover, even if VKORC1L1 is expressed at a lower level than VKORC1 in the adult liver, these data suggest that it can contribute to some extent to the production of functional coagulation factors by the liver, when VKORC1 is fully inhibited, at least for a few days. In summary, additional work will be needed to identify the warfarin resistant enzyme(s) responsible for the antidotal effect of vitamin K in vivo.

## 13. Conclusions

VKORC1 and its paralog, VKORC1L1, were identified almost 15 years ago. Understanding of the biochemistry, genetics and physiology of VKORC1 has tremendously progressed since then, although the characterization of its paralog has only been initiated in the last few years. It is now clear that VKORC1L1 is a functional vitamin K oxidoreductase, which can support the vitamin K-dependent carboxylation of proteins in cell culture and in vivo, at least when VKORC1 is inactivated. VKORC1L1 is less sensitive to warfarin than VKORC1, although the two enzymes appear to be inhibited by this drug through similar mechanisms, which is consistent with the conclusion that they also share a very similar structural organization. A key question that remain unanswered regarding VKORC1L1 biology is whether its function in vivo is to support vitamin K reduction only in the context of γ-carboxylation, or if it has any additional physiological roles. Future research, taking advantage of recently developed mouse models, will be focused on addressing this question. For instance, the careful phenotypic characterization of the *Vkorc1l1*^−/−^ mice should provide important clues to identify the unique functions of VKORC1L1 in vivo.
